# Effective Connectivity for Decoding Electroencephalographic Motor Imagery Using a Probabilistic Neural Network

**DOI:** 10.3390/s21196570

**Published:** 2021-09-30

**Authors:** Muhammad Ahsan Awais, Mohd Zuki Yusoff, Danish M. Khan, Norashikin Yahya, Nidal Kamel, Mansoor Ebrahim

**Affiliations:** 1 Centre for Intelligent Signal & Imaging Research (CISIR), Electrical & Electronic Engineering Department, Universiti Teknologi PETRONAS, Seri Iskandar 32610, Perak, Malaysia; mzuki_yusoff@utp.edu.my (M.Z.Y.); danish_mkhan@yahoo.com (D.M.K.); norashikin_yahya@utp.edu.my (N.Y.); nidalkamel2@hotmail.com (N.K.); 2 Department of Telecommunications Engineering, NED University of Engineering and Technology, Karachi 75270, Pakistan; 3 Faculty of Engineering, Sciences, and Technology, Iqra University, Karachi 75500, Pakistan; mebrahim@iqra.edu.pk

**Keywords:** brain–computer interface, brain effective connectivity, PDC, DTF, PhysioNet motor imagery, probabilistic neural network, SVM, KNN, decision tree

## Abstract

Motor imagery (MI)-based brain–computer interfaces have gained much attention in the last few years. They provide the ability to control external devices, such as prosthetic arms and wheelchairs, by using brain activities. Several researchers have reported the inter-communication of multiple brain regions during motor tasks, thus making it difficult to isolate one or two brain regions in which motor activities take place. Therefore, a deeper understanding of the brain’s neural patterns is important for BCI in order to provide more useful and insightful features. Thus, brain connectivity provides a promising approach to solving the stated shortcomings by considering inter-channel/region relationships during motor imagination. This study used effective connectivity in the brain in terms of the partial directed coherence (PDC) and directed transfer function (DTF) as intensively unconventional feature sets for motor imagery (MI) classification. MANOVA-based analysis was performed to identify statistically significant connectivity pairs. Furthermore, the study sought to predict MI patterns by using four classification algorithms—an SVM, KNN, decision tree, and probabilistic neural network. The study provides a comparative analysis of all of the classification methods using two-class MI data extracted from the PhysioNet EEG database. The proposed techniques based on a probabilistic neural network (PNN) as a classifier and PDC as a feature set outperformed the other classification and feature extraction techniques with a superior classification accuracy and a lower error rate. The research findings indicate that when the PDC was used as a feature set, the PNN attained the greatest overall average accuracy of 98.65%, whereas the same classifier was used to attain the greatest accuracy of 82.81% with the DTF. This study validates the activation of multiple brain regions during a motor task by achieving better classification outcomes through brain connectivity as compared to conventional features. Since the PDC outperformed the DTF as a feature set with its superior classification accuracy and low error rate, it has great potential for application in MI-based brain–computer interfaces.

## 1. Introduction

Many unfortunate people with severe motor disabilities are not able to communicate well with the outside world. Their disabilities become obstacles between them and their social lives. Millions of people around the globe are affected by these types of disabilities, which are caused by several medical conditions, such as trauma, stroke, and different neurodegenerative diseases, including Alzheimer’s disease (AD), Parkinson’s disease (PD), motor neuron diseases (MND), etc. Such people are afraid to be neglected as a significant part of society. Research communities around the world are working on the development of brain–computer interfaces based on different medical applications, such as brain-controlled wheelchairs, thus helping patients who have lost their abilities to communicate and providing them with mobility.

**Figure 1:**
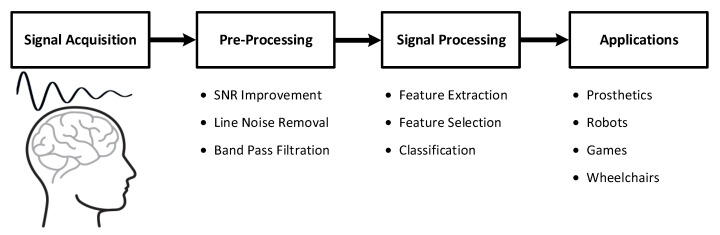
General overview of brain–computer interface (BCI) systems.

The brain–computer interface (BCI) has been one of the most rapidly growing technologies in recent years. It provides a control system that is capable of transforming a user’s intentions into special commands to be used as a communication bridge between the brain and the outside world [1,2]. BCIs are a combination of software and hardware technologies that allow the brain to control external devices, such as prosthetic arms/legs or wheelchairs, by decoding different brain patterns. A general representation of a BCI system is given in Figure 1.

Electroencephalography (EEG) is the conventional method of monitoring the electrical activities of the brain by placing special sensors called electrodes on the surface of the scalp [3]. Electrical signals are generated by the inter-communication of cells within the brain. EEG-based BCIs identify explicit frequency patterns in the brain by sensing slight variations in the voltages that the brain emits while the person thinks in any way.

Motor imagery (MI) a traditional and active BCI paradigm that uses electroencephalography (EEG) to directly reflect a user’s intention. Motor imagery can be expressed as the process of performing the imagination of motor tasks (i.e., the movement of body parts) without actually executing them physically [4].

Although the literature shows positive outcomes and accomplishments by using conventional MI-based BCI systems, including different state-of-the-art feature extraction and classification techniques, there are still many barriers and hurdles in using the technology efficiently and effectively. The major drawback of the existing MI-based BCIs is that they are based on traditional feature extraction and classification algorithms. Traditional feature extraction methods use MI-responsive frequency bands that do not have inter-subject or intra-subject consistency, which creates instability in BCI systems [5]. ERS/ERD analysis has proven to be complex due to its occurrence in different parts of the brain, during different time intervals, and at different frequencies, thus making it difficult to obtain significant features for classification [6]. Considering the low amplitude and noisy nature of EEG data, pattern inconsistency among multiple subjects and even altered patterns within a session with the same subjects can be expected. Various EEG studies confirmed the occurrence of MI-actuated signals in primary sensorimotor areas [7,8], whereas other researchers also reported the inter-communication of multiple brain parts during cognitive tasks [9,10], thus making it difficult to isolate one or two regions where the activity takes place. Furthermore, conventional MI-based BCI systems utilize temporal–spectral features from individual channels to recognize motor imagery patterns, which may not provide sufficient information. Therefore, a deeper understanding of the behavior of the brain’s neural patterns is important in order to provide more useful and insightful features for BCIs, since the execution of motor or cognitive tasks results in the exchange of information of multiple mutually interconnected brain regions. Thus, awareness of brain connectivity has become a key aspect of neuroscience and of understanding the behaviors of different regions. Different MI tasks are expected to have associations with particular brain connectivity patterns among the brain regions. Therefore, brain connectivity provides a promising approach to solving the stated shortcomings by considering inter-channel/region relationships during motor imagination. EEG recordings can be used to identify these connectivity patterns and offer unique features to infer a subject’s intentions.

Several conventional classification algorithms have been widely used in brain- connectivity-based BCIs. In a study by Mehdi et al. [11], MVAR (multivariate autoregressive)-based source localization was used with an SVM for the classification of MI tasks, whereas Liang et al. [12] used the combination of the PDC and MEMD with an SVM to classify two-class MI. In another work [13], Panche et al. used a linear discriminant analysis (LDA) classifier for the prediction of MI tasks using the transfer-entropy-based effective connectivity. Lingyun et al. [14] used brain network analysis for the classification of lower-limb motor imagery; the authors used a sparse multinomial logistic regression (SMLR)-based SVM for the prediction of the MI. In the work of Rahman et al. [15], an fNIRS-based BCI was proposed by using effective temporal window estimation. The extracted features were used with three classifiers—LDA, SVM, and KNN—for the prediction of two-class MI.

Human brain mapping has primarily been used to construct maps that indicate regions of the brain that are activated by certain tasks. The term brain network or brain connectivity refers to sets of interconnected brain regions among which information is transferred. However, there has been insufficient discussion about the use of brain connectivity for motor-imagery-based pattern recognition. Our research is based on the analysis of effective connectivity, which explains the effects of neurons on each other, thus representing the causal connections between activated brain regions. The technique proposed in this study is based on brain connectivity, which is a unique direction for research on building a more accurate framework. The accuracy and performance improvements of the developed system in this work will be a step forward for the effective implementation of BCI applications, such as brain-controlled wheelchairs.

The rest of this paper is structured as follows: Section 2 describes the use of brain connectivity in the field of motor-imagery-based brain–computer interfaces. Subsequently, Section 3 covers the detailed description of an MI-based EEG dataset. Section 4 discusses the methods used in this work, including the preprocessing, feature extraction, and classification techniques. It further describes the detailed estimation of the PDC and DTF, along with the description of the evaluation measures. Section 5 presents the results and discussion. Finally, Section 6 concludes the paper.

## 2. Related Work

The study of brain connectivity is based on three distinct but related types of connectivity, including anatomical connectivity (AC), functional connectivity (FC), and effective connectivity (EC) [16,17]. Connectivity patterns are created by structural connections, such as synapses or fiber pathways, or they exemplify statistical or causal relationships, which are measured as cross-correlations, coherence, or information flow [18,19]. Among the different types of feature representations for motor-imagery-based EEG decoding, the connectivity models of multi-channel signals may produce more discriminating features for significant classification [20,21]. Several approaches to analyzing motor-imagery-based BCI systems that were established using brain connectivity have been proposed in the last few years.

Billinger et al. [22] proposed a technique for obtaining single-trial directed transfer functions (DTFs) by using vector-autoregressive (VAR) independent variable models for MI-based BCI classification, and the classification findings were identical to the band-power (BP) characteristics. Ming et al. [23] researched EEG characteristics associated with the movement of the left and right fingers. The event-related desynchronization (ERD) and movement-related cortical potential (MRCP) features were recovered using common spatial patterns (CSP) and discriminative canonical pattern matching (DCPM). Since pre-movements have supportive MRCP and ERD characteristics, the proposed DCPM and CSP combination approach may be able to recognize them effectively. Mehdi et al. [11] proposed a method in which they used an MVAR model with the source localization algorithm (sLORETA) to extract active sources. After incorporating ANOVA for the reduction of the feature set, the authors used an SVM for the classification of the motor imagery tasks. In another study [24], a new time- and frequency-based causality was proposed by using a time-invariant BVAR model to investigate the flow of causality in the central region of the brain. As a result, improved performance with the new causality (NC) was reported as compared to the Granger causality.

In Rathee et al.’s study [25], the time-domain partial Granger causality (PGC) in terms of the connectivity feature set was used in an MI-based BCI environment. This resulted in the improved discriminability of MI tasks by using a single-trial effective connectivity distribution. However, Yang et al. [20] proposed a method in which time- and frequency-conditional Granger causality (CGC) was determined using a regularized orthogonal forward regression (ROFR) algorithm. The extracted features were classified using a boosted convolutional network, resulting in enhanced classification accuracy. Ahmad et al. [26] proposed an effective connectivity analysis for MI by using several variants, including DTF, direct DTF, and generalized PDC. A hierarchical feature selection technique was adopted to select the most important connectivity features, which resulted in successful discriminations of mental arithmetic tasks. Short-term DTF was used to investigate brain activities by implementing it to evaluate motor imagery experiments in three channels—C3, C4, and Cz [27,28]. Liang et al. [12] explored the effective connectivity in the motor cortex by using a combination of the PDC and multivariate empirical mode decomposition (MEMD). The results demonstrated the existence of significant effective connectivity in the bilateral hemisphere during the MI tasks.

However, in a study by Chung et al. [29], the temporal patterns of connectivity among EEG channels were evaluated according to the time-varying patterns of the channel-to-channel correlation coefficients and the average correlation coefficients per channel for left- and right-hand motor imagery. In [30], Lee et al. predicted the MI performance by using dynamic causal modeling to study the connectivity of a rest-state network, which affected the performance of the MI. As a result, a significant difference was observed in the network strength from the motor cortex to the right prefrontal cortex between the high- and low-MI-performance groups. Independent-source-based causal brain connectivity was introduced in [31] for the classification of left- and right-hand motor imagery. Chen et al. [32] used Granger causality analysis to analyze the brain connectivity between the motor, contralateral premotor, and sensorimotor areas. The results also revealed the significant difference in the G-causality trial numbers of left- and right-finger motor imagery. Li et al. [33] investigated effective connectivity in order to analyze and compare the rest state with right-hand motor imagination. In another study [13], Panche et al. proposed Renyi-based transfer entropy for measuring effective connectivity, resulting in significant robustness against varying amounts of data and noise levels.

## 3. Dataset Description

### 3.1. Ethical Approval

The dataset used in this work was entirely de-identified; therefore, no ethical review board (ERB) permission was required. The publicly accessible Physionet EEG motor imagery dataset used in this research work is available online [34], and it can be used without any further authorization.

### 3.2. Dataset

The developers of the BCI2000 instrumentation system created the dataset used in this work. The database includes more than 1500 one- and two-minute recordings from 109 volunteer subjects. The overall dataset contains the recordings of both the actual and the imagined motor tasks from all of the participants [35].

Each subject underwent 14 runs, where they performed several tasks, including the actual movement of the right/left fist, the imagination of the movement of the right/left fist, the actual movement of both fists/both feet, the imagination of the movement of both fists/both feet, and the opening/closing the eyes. EEG recordings were obtained as per the international 10–10 system from 64 electrode channels (excluding Nz, F9, F10, FT9, FT10, A1, A2, TP9, TP10, P9, and P10) that were sampled at the rate of 160 samples per second.

**Figure 2:**
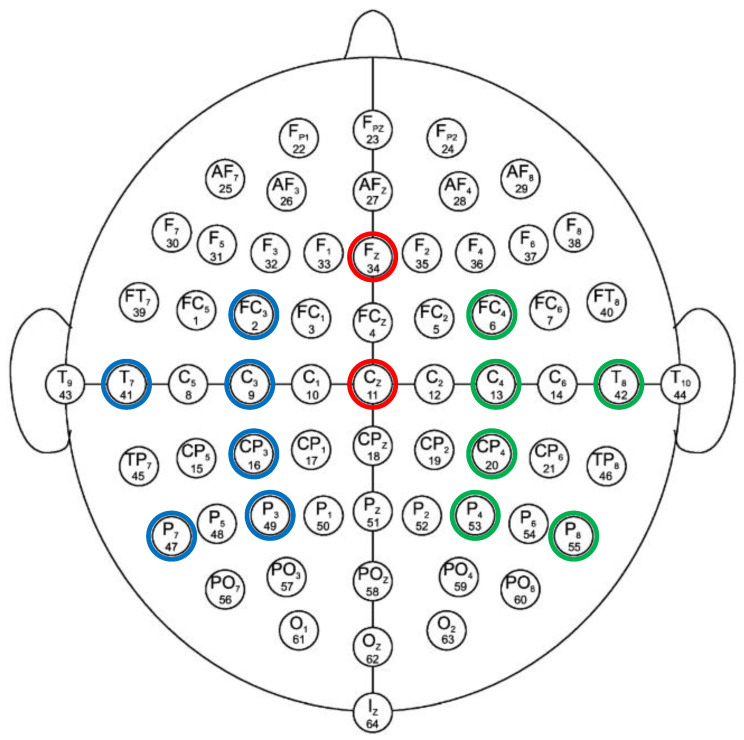
Standard 10–10 EEG electrode configuration. Selected electrodes from left, right and central region are encircled with blue, green and red color, respectively.

For the purpose of analysis, 14 out of the 64 EEG electrodes from all of the major regions of the brain (left region, right region, and central region) were used in this study. Among these 14 carefully chosen channels, two electrodes (i.e., Cz and Fz) are in the central region of the brain, six electrodes (i.e., T7, P7, C3, P3, FC3, and CP3) are located in the left-brain region, and the remaining six electrodes (i.e., T8, P8, C4, P4, FC4, and CP4) are in the right region of the brain. These selective electrodes are capable of analyzing the effective network during the MI-demonstrated brain networks underlying the mental behavior in several brain parts, including the primary motor cortex, primary and secondary somatosensory cortices, lateral premotor cortex, supplementary and presupplementary motor areas, frontal cortex, temporal cortex, and parietal cortex [36,37]. These are the most suitable electrodes for the feasible implementation of MI-based BCI using multiple brain regions with a reduced number of electrode channels. Moreover, these channels have been endorsed by several researchers in their work [38,39,40,41,42]. The channels chosen for the BCI application are shown in Figure 2.

In this work, we excluded the data from 18 subjects—namely, S29, S30, S34, S37, S41, S51, S64, S72, S73, S74, S76, S88, S89, S92, S100, S102, S104, and S106—since they had contaminated EEG recordings or an insufficient number of samples in the available dataset. Thus, the EEG data from 91 out of the 109 subjects were used in this study. Among the several tasks available in the stated database, two-class motor imagery (imagination of opening/closing the left fist and imagination of opening/closing the right fist) was analyzed.

### 3.3. MI Paradigm

**Figure 3:**
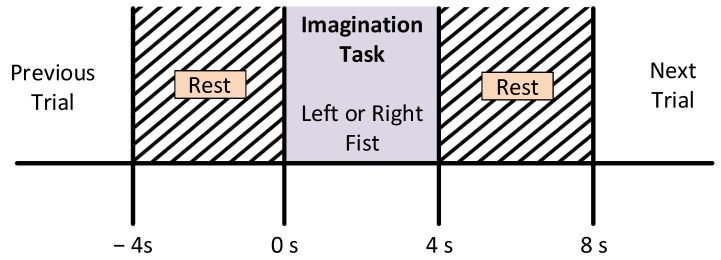
Motor imagery time scheme during the recording of the EEG signals.

The subjects were asked to sit on a comfortable armchair in front of a screen in order to guide them through the experimental procedure. They were instructed not to move any parts of the body during the recording of the data. For the experiment, participants were instructed to perform the imagination of motor tasks as a target appeared on either the left or the right side of the screen. The subjects imagined opening and closing the corresponding fist until the target disappeared. Then, the subject relaxed. Every subject recorded three sessions for each type of MI task. However, a single session comprised seven to eight random trials of each class, i.e., left or right movement imagery. Each trial was carried out for four seconds, followed by a rest period of 4 s ± 5%, as shown in Figure 3.

## 4. Methodology

The proposed methodology aimed to utilize the estimation of brain connectivity (effective connectivity) for the classification of motor-imagery-based electroencephalographic signals using different classifiers. To the best of the authors’ knowledge, a probabilistic neural network has never been used for the task of MI prediction using brain connectivity. This study provides a comparative analysis of all of the traditional and unique classifiers for two-class MI classification by using connectivity features.

**Figure 4:**
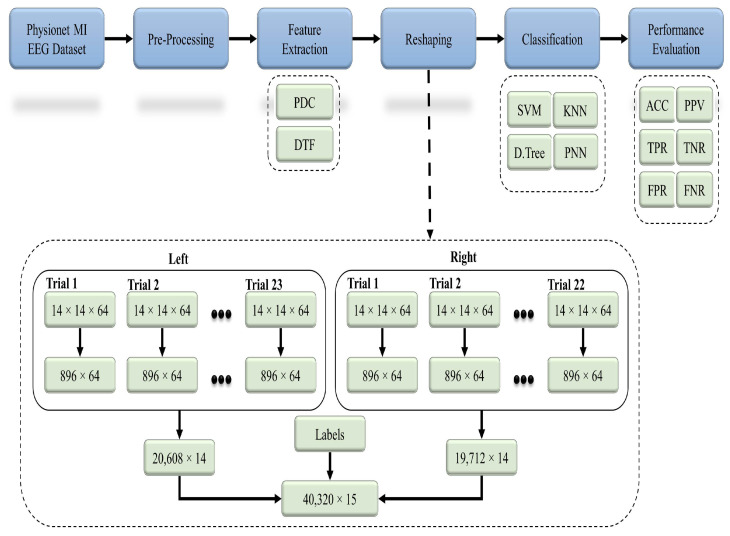
General workflow diagram for the classification of the two-class MI EEG using the effective connectivity matrices with the PDC and DTF.

Figure 4 represents the flow of the proposed methodology, in which raw EEG data from the 91 healthy subjects were preprocessed with different techniques. Based on the studies from the literature, 14 significant channels were selected out of a total 64 channels, and their effective connectivity in terms of the partial directed coherence (PDC) and directed transfer function (DTF) was used as a key feature. Both the PDC and DTF were analyzed separately by using the classifiers in order to recognize the 2-class MI-based patterns.

The proposed work was implemented on Intel^®^ Xeon(R) CPU E3-1226 v3 at 3.30 GHz (installed memory: 16 GB). MATLAB 2020a was used as the programming platform for our proposed work.

### 4.1. Preprocessing

The enhancement of the raw signal is the prerequisite and fundamental step of EEG signal processing. The raw data hold both significant information and artifacts/undesired components (i.e., eye blinks, eyeball movement, jaw clenches, heavy breaths, etc.). The exclusion of unnecessary components from the signal is very important in order to improve the signal-to-noise ratio (SNR).

The data from the 91 subjects were preprocessed using Brainstorm in MATLAB. Brainstorm is an open-source platform dedicated to the analysis of several types of brain recordings, such as fNIRS, MEG, ECoG, and EEG. A high-pass filter was applied at 0.1 Hz for the purpose of DC offset correction, while a notch filter was used at 60 Hz to eliminate the electrical interference. The raw signal was bandpass filtered between 7 and 32 Hz to exclude all of the frequency components other than mu and beta, as the studies [43,44,45] revealed the occurrence of MI patterns in the stated frequency range. However, the artifacts were removed using the EEGLAB-based artifact removal algorithm called artifact subspace reconstruction (ASR). Major artifacts identified by the ASR technique, including eye blinks, muscle noise, and sensor motions, were removed from the data. Furthermore, excessive preprocessing was avoided in this research, since the creator of the DTF/PDC suggested that unnecessary preprocessing may impact the causality information and should, thus, be avoided [46].

Although the data were preprocessed to remove the artifacts and unnecessary signals, there was still redundant information available, which was also eliminated. These redundant data were actually the duration during the trial when the participants rested and did not perform any type of MI task (see Figure 3). After discarding the undesired part of the MI trials, 4095 trials (4 sec each) were available for further signal processing. Since the sampling frequency was 160 Hz, the total number of samples for each trial was equal to 4×160=640. In this work, we used 14 significant channels, as discussed in the dataset description.

### 4.2. Feature Extraction

The next critical step of the signal processing of the BCI system after preprocessing was feature extraction. This process was intended to extract specific characteristics of the signals that encoded the messages or commands elicited in the user’s brain by either evoked or spontaneous inputs. In this work, effective connectivity was estimated using the partial directed coherence (PDC) and directed transfer function (DTF) for the MI-based EEG classification.

The basic code for the calculation of the PDC and DTF is available at [47]. The PDC and DTF were calculated by adjusting the maximum frequency to 32 Hz, whereas the number of bins was set to 64. Both the PDC and DTF were calculated for every 4 s of EEG data, which referred to a single MI trial (see Figure 3). Every subject recorded 23 trials for the left direction and 22 trials for the right direction. The feature sets calculated for each trial were in the form of 3D matrices (i.e., 14×14×64), which were converted into 2D (i.e., 896×14) by performing matrix reshaping in order to execute the classification process. For the left class, each of the 23 trials (896×14) was concatenated to get a (20,608×14) matrix, whereas for the right class, each of the 22 trials of (896×14) was concatenated to get a (19,712×14) matrix. To create the final feature set, both the left and right feature sets were combined along with an extra column (i.e., labels for both classes) to get a (40,320×15) matrix. This final feature set for each subject set was used as an input for different classification algorithms for the 2-class MI prediction (see Figure 4).

#### 4.2.1. Effective Connectivity

Effective connectivity can be interpreted as the indirect or direct influence of one neural system on another at either a synaptic level or a cortical level [48]. According to [49], the EC should be recognized as the time-dependent and simplest possible circuit diagram that replicates the timing relationships between the recorded neurons. There are several brain connectivity estimators, including non-linear estimators, linear estimators, bivariate estimators, and multivariate connectivity estimators. The difference between bivariate and multivariate estimators is presented in [50], which states that the presence of multiple channels (i.e., more than two) in the case of an interrelated system of channels in bivariate connectivity estimators provides erroneous information due to the electrode channels positioned at different distances causing notable delays in the recorded signal.

This fact has led the research community to consider multivariate estimation for effective connectivity. These multivariate estimators based on Granger causality (GC) [51,52] allow precise measurement of the directed connectivity by eliminating the problem caused by multiple channels in the bivariate method. The Granger causality (GC) was established to determine the causal connection between two signals. If past information of signal X(t) is given with the past information of signal Y(t), then the causality between X(t) and Y(t) can be measured by decreasing the error (prediction error) of signal Y(t).

The partial directed coherence [53] and directed transfer function [54] are among the most widely used connectivity estimators based on the multivariate autoregressive model (MVAR) under the umbrella of Granger causality (GC) to evaluate the directional influences of any given pair of channels in a dataset. The PDC and DTF, which are based on the MVAR model, can detect causal interactions between the signals and identify the directional propagation of the EEG activity in terms of the frequency function. The frequency dependency of estimators is an essential aspect, since various EEG rhythms play different roles in the processing of information. The PDC and DTF are insensitive to volume conduction and are very tolerant towards noise, as they are based on the phase differences between channels of multivariate data. The physiological information provided by means of the parametric and multivariate autoregressive (MVAR)-based methods has revealed their effectiveness in brain research.

#### 4.2.2. Partial Directed Coherence

Baccala and Sameshima [53] developed an analysis method called partial directed coherence (PDC) as an extension of Granger’s conditional causality in the frequency domain. The analysis aims to set up the connectivity links among different brain regions for the frequency range selected from an electroencephalographic signal. In this work, we selected two frequency ranges, commonly known as alpha (7–13 Hz) and beta (13–32 Hz).

The multi-channel EEG data obtained by using multivariate autoregressive (MVAR) model can be defined as follows:

(1) $$Y\left(t\right)=\sum_{r=1}^{l}A\left(r\right)Y\left(t-r\right)+E\left(t\right),$$

where Y(t) denotes the 14-channel EEG time-series data, *l* is the model order, A(r) is the coefficient matrix with lag *r*, and E(t) is the error (prediction error) of the multivariate autoregressive model. Equation (1) can be rearranged to determine the prediction error as follows:

(2) $$E\left(t\right)=\sum_{r=0}^{l}\hat{A}\left(r\right)Y\left(t-r\right),$$

where the following value belongs to $\hat{A}\left(r\right)$:

(3) $$\hat{A}\left(r\right)=\left\{\begin{array}{cc} 1-A(r), & r=0.\\ -A(r), & r>0. \end{array}\right.$$

The error function in Equation (2) can be expressed in the frequency domain:

(4) E(f)=A(f)Y(f),

where *f* represents the frequency. So,

(5) $$A\left(f\right)=\sum_{r=0}^{l}\hat{A}\left(r\right)\exp{}^{-j2\pi fr}.$$

The PDC in the frequency domain can be calculated as

(6) $$P_{ij}\left(f\right)=\frac{A_{ij(f)}}{\sqrt{\sum_{k=1}^{x}{\left|a_{kj}{\left(f\right)}^{2}\right|}^{2}}}.$$

In Equation (6), the number of analyzed channels (except for the current channel *j*) is denoted by *x*, while $P_{ij}$ represents the PDC’s correlation indicators from $Y_{j}$ to $Y_{i}$ at a specific frequency *f*. The capital *A* represents the whole coefficient matrix; however, the small *a* refers to the matrix elements. The sum of all causal inference estimations $P_{ij}$ at certain frequencies while reviewing the influence on all channels *x* by channel *j* is 1. This proves that higher values of $P_{ij}$ result in a greater influence of channel *j* on channel *i*.

#### 4.2.3. Directed Transfer Function

Kaminski and Blinowska [54] presented an analysis method based on a multivariate model called the directed transfer function (DTF). This work generalized Granger’s work to some extent and claimed to have significant superiority in brain connectivity estimation. The PDC and DTF differ in such a way that the PDC detects active direct directional coupling, while the directed transfer function illustrates the presence of both direct (i.e., the immediate causal influence path) and indirect (the signal traveling through intermediate structures rather than an instant direct causal influence path) directional signal propagation [55].

The only different step in the DTF is that of taking the inverse of A(f) from Equation (5) and then performing the normalization.

(7) $$H\left(f\right)=A{\left(f\right)}^{-1}$$

where H(f) is the frequency-domain representation of the transfer function of the system and can be obtained as follows:

(8) $$H\left(f\right)=\frac{1}{1+\sum_{r=0}^{l}\hat{A}\left(r\right)\exp{}^{-j2\pi fr}}.$$

Then, the squared DTF from channel *j* to *i* can be given as

(9) $$D_{i\leftarrow j}^{2}=\frac{|H_{ij}{\left(f\right)|}^{2}}{\sum_{m=1}^{y}{\left|H_{im}\left(f\right)\right|}^{2}}$$

(10) $$D_{i\leftarrow j}=\frac{|H_{ij}\left(f\right)|}{\sqrt{\sum_{m=1}^{y}{\left|H_{im}\left(f\right)\right|}^{2}}}$$

Here, $D_{i\leftarrow j}$ represents the normalized version of causality from channel *j* by channel *i* at some specific frequency *f*, while the transfer matrix of the multivariate autoregressive model is denoted by $H_{ij}$.

#### 4.2.4. Connectivity Estimation

There were several steps (i.e., dataset adjustment, model order calculation, MVAR coefficient determination, and PDC/ DTF estimation) involved in the estimation of effective connectivity.

1. The first step in the estimation of connectivity was to adjust the MI EEG dataset by selecting the significant electrode channels from the primary dataset. The selection of 14 channels was already discussed in the preprocessing section.
2. After selecting the number of channels for the connectivity estimation, the data were divided into several trials, and the connectivity was computed separately for each trial.
3. Next critical step was the calculation of the model order *l*, which defined how many previous samples were needed for the prediction of the current samples. This was an automatic process that required a minimum and maximum range of order (i.e., 1–20 in our case) and an optimizing algorithm (i.e., Schwarz’s Bayesian Information Criterion in our case) to select the order with the minimum error. However, the model order *l* was calculated by using the ARFIT toolbox with the parameters suggested by several researchers [56,57,58].
4. After estimating the optimized model order *l*, the next step incorporated the estimation of the MVAR coefficients (see Equation (1)).
5. The next step was to define the sampling frequency (i.e., 160 Hz) and the number of frequency bins among which the total frequency range (i.e., 7–32 Hz) would be divided for the connectivity analysis. In this work, we set the number of frequency bins to 64 so that the connectivity estimation process would be repeated 64 times for each bin of the frequencies.
6. The next step after the assignment of the above parameters was to find the difference $\hat{A}$ by subtracting the MVAR coefficient matrix *A* from the identity matrix *I*, as in Equation (3).
7. After calculating the difference from the identity matrix, a Fourier transform was performed to convert the time-series MVAR matrix $\hat{A}$ into the frequency domain A(f) (see Equation (5)).
8. The estimation of both the PDC and DTF followed all of the above-mentioned steps; however, for the DTF, the only different step was to find the inverse of the frequency domain matrix (i.e., $H\left(f\right)=A{\left(f\right)}^{-1}$), where *H* is called the transfer matrix of the system (see Equation (7)).
9. The final step in the estimation of the connectivity was the normalization of A(f) and H(f) for the PDC and DTF, respectively (see Equations (9) and (10)). The normalized outputs *P* and *D* were then called the PDC and DTF, respectively.
10. The 14-channel data were used while incorporating 64 frequency bins; therefore, the estimation of the PDC and DTF resulted in a 14×14×64 matrix for each trial. Since the estimated connectivity matrix was in 3D, matrix reshaping was carried out to convert the 3D matrix into a 2D matrix for the purpose of classification.

### 4.3. Classification

Classification is a mechanism by which target variables or classes are predicted from given information. An extensive motor-imagery-based EEG dataset covering 91 healthy subjects was used for the estimation of brain connectivity with measures of the effective connectivity (i.e., PDC and DTF). The extracted features in terms of the DTF and PDC were classified by using four classifiers to predict 2 classes of left/right EEG MI. The k-fold cross-validation (CV) technique was used to explore the performance of the proposed method, and k = 5 and 10 were used for all experiments, as they were found to be appropriate.

K-nearest neighbors (KNN) belongs to the category of supervised learning, and it can be used for both classification and regression problems. In KNN, the item is identified by a majority vote from its neighbors, with the item being allocated to the most common class of its nearest k neighbors [59]. In this work, standardized data were used in KNN with the Euclidean distance function while using equal-distance weights, and the number of neighbors was set to 3.

Support vector machine (SVM) is a supervised-learning-based classification model that is explicitly described by a separating hyperplane [60]. This work aims to use a 2-class SVM with a Gaussian kernel function for the classification of MI-based EEG data. Standardized data were used in the stated variant of the SVM with a kernel scale of 0.9399 and a box constraint of 1.

The decision tree is a non-parametric predictive modeling approach to supervised machine learning that covers both classification and regression problems. As the name indicates, it utilizes a tree-like structure of decisions. In the tree model, class labels or final outcomes are represented by the leaves, while the decision nodes contain the split data [61]. Decision trees are significantly easy to interpret and simple to implement.

#### Probabilistic Neural Networks (PNNs)

A probabilistic neural networks (PNN) is a supervised approach that excels in decision making and classification. The network is trained with objects from known classes by using a collection of known instances, and thereafter, it can distinguish new objects according to the categories specified in the training set [62].

**Figure 5:**
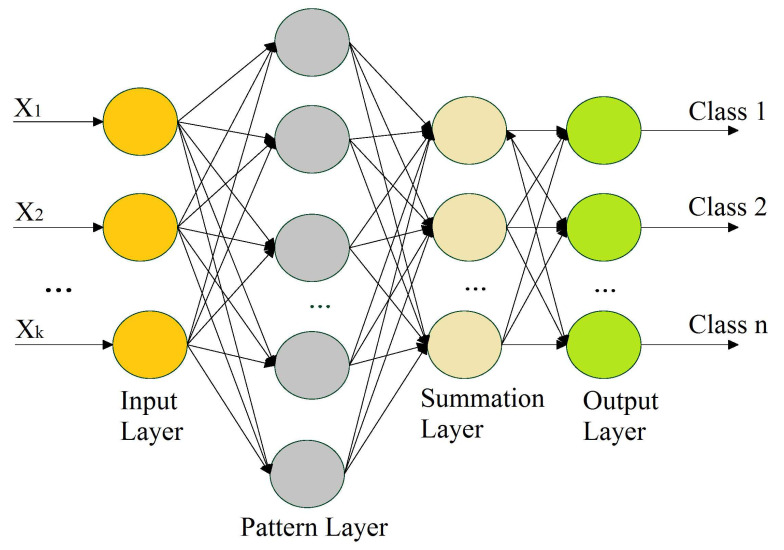
Illustration of a probabilistic neural network.

A PNN is a feedforward neural network that is commonly used in classification problems, and it does not require the extensive forward and backward calculations used in traditional neural networks. A PNN is intimately associated with the estimation of the Parzen window probability distribution function (PDF). This comprises several networks, where each sub-network is a Parzen window PDF estimator for each class [63]. A PNN is a feedforward multilayered network with four layers—an input layer, pattern layer, summation layer, and output layer—as shown in Figure 5.

The 1st layer performs the distribution of the input values from the input layer to the neurons of the 2nd layer, i.e., the pattern layer. The pattern layer incorporates pattern units that are equal to the number of samples. Each pattern unit performs a two-step process on the input, including the computation of the Euclidean distance and the implementation of the kernel function, which computes the output when the pattern *x* is received from the input layer. The calculations from the pattern layer are passed to the 3rd layer (i.e., summation layer). The number of neurons in the 3rd layer is equal to the number of available classes, where each neuron is linked to all of the neurons in the pattern layer associated with the class represented by the particular unit. Each neuron represents the probability of the pattern *x* that is classified. The summation layer also determines the false detection of any given class. Weighted votes or values for each class from the 3rd layer are presented to the decision layer, where the majority voting is carried out in order to compare the values for each class, and the highest value predicts the target class.

The training process is perhaps the most time-intensive element of the preparation of a network for usage, since it depends on iterative algorithms that define the weight values. There are three stages in which the weight definition process occurs. In the 1st stage, weights are defined between the input and pattern layer. The definition of weights is instantaneous. As a result, training is accomplished by showing the training set examples all at once (after normalization) and sharing the node values for the layer of examples. In the 2nd stage, weights are predetermined between the pattern and summation layers and are equal to 1. In the 3rd stage, the weights are preset between the summation and output layers; however, their values may be given based on certain factors according to the training samples [62]. In this work, the spread of 0.04 was used as a hyperparameter for the proposed PNN model. This parameter was rigorously tuned to achieve consistent results.

### 4.4. Evaluation Parameters

The performance of the proposed work was determined by using several evaluation parameters. The evaluation parameters incorporated in this work are given below:

1. Classification accuracy (*CA*):
  (11) $$CA=\frac{TP+TN}{TP+TN+FP+FN}$$
2. Sensitivity or true positive rate (*TPR*):
  (12) $$TPR=\frac{TP}{TP+FN}$$
3. Specificity or true negative rate (*TNR*):
  (13) $$TNR=\frac{TN}{TN+FP}$$
4. Precision or positive predictive value (*PPV*):
  (14) $$PPV=\frac{TP}{TP+FP}$$
5. False positive rate (*FPR*):
  (15) $$FPR=\frac{FP}{FP+TN}$$
6. False negative rate (*FNR*):
  (16) $$FNR=\frac{FN}{FN+TP}$$

Here, TP, FP, TN, and FN represent true positives, false positives, true negatives, and false negatives, respectively.

### 4.5. Statistical Investigation

A multivariate analysis of variance (MANOVA) pairwise comparison was performed with a significance threshold of 0.05 to determine the statistical significance in the features of the left and right motor imagery EEG. For this, the 2 classes (left and right) were treated as fixed factors, while connections from all of the subjects were used as dependent variables. The mean difference between the 2 classes for each connection was tested with a 95% confidence interval, and the connection was marked as significant if the *p*-value was less than 0.05. As an adjustment for multiple comparisons, Bonferroni correction was implemented.

## 5. Results and Discussions

In this work, motor-imagery-based pattern recognition was experimented upon by using effective connectivity features, the PDC, and the DTF. The effective connectivity among three different brain portions—the central, left, and right regions—was measured using 14 electrode channels (i.e., 196 connectivity pairs) for which statistical analysis was carried out in order to determine the significant connections. The performance of the proposed work was evaluated separately for each subject; however, the results are presented as the averages of all 91 subjects.

### 5.1. Statistical Analysis

**Figure 6:**
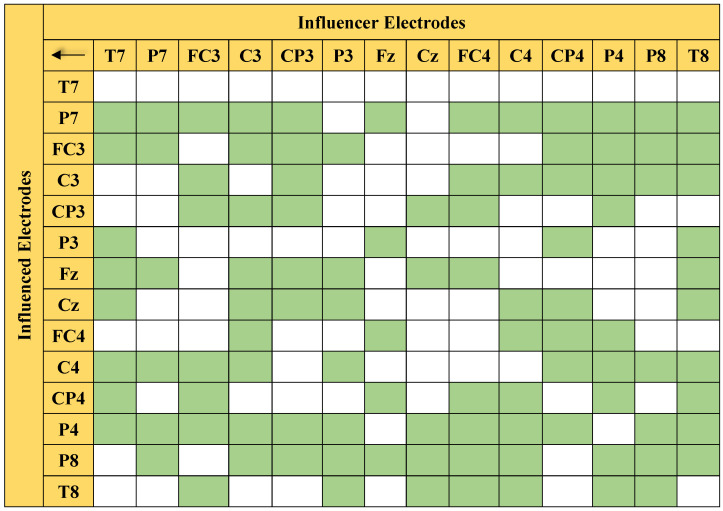
Matrix-form illustration of 105 significant connections (in green) listed in Table 1.

Figure 6 presents the connectivity pairs with significant differences that were obtained through MANOVA. The green color highlights the significant connections, whereas the white boxes indicate the insignificant pairs.

The statistical analysis showed that 105 out of the 196 connections were identified as significant with a 95% confidence level. With more than half (55.6%) of the connections at the 95% confidence level, the classification of the left/right MI EEG based on the 14 electrodes is expected to give good performance. Table 1 provides the *p*-value of the significant pairs. As described in Section 4.5, a *p*-value that is less than 0.05 basically indicates that the difference between the classes of the said connections is significant. This implies that the significant connections are dominant PDC features that largely contribute to the high classification accuracy of the proposed method.

### 5.2. Classification of the MI EEG Using the EC

In this research work, two cases (with respect to the feature set and the classifier) were examined for the classification of the two-class motor imagery EEG recordings. A description of each case is given below.

- **Case 1:** The partial directed coherence (PDC) was used as a feature set with four classifiers: SVM, decision tree, KNN, and PNN.
- **Case 2:** The directed transfer function (DTF) was used as a feature set with the four classifiers stated in Case 1.

In Case 1, the PDC with SVM provided average classification accuracies of 96.30% and 97.45% for 91 subjects when using 5- and 10-fold cross-validation (CV), respectively. The PDC with KNN resulted in mean CAs of 97.85% for 5-fold CV and 98.63% for 10-fold CV. The PDC with the decision tree resulted in average CAs of 63.85% and 64.92%, whereas the average accuracies for the PDC with the PNN were 97.87% and 98.65% for 5- and 10-fold cross-validation, respectively. The classification accuracies and other evaluation parameters for Case 1 are presented in Table 2.

In Case 2, the DTF with SVM provided average classification accuracies of 81.83% and 82.69% for 91 subjects when using 5- and 10-fold cross-validation (CV), respectively. The DTF with the KNN resulted in mean CAs of 82.04% for 5-fold CV and 82.67% for 10-fold CV. The DTF with the decision tree resulted in average CAs of 61.42% and 61.95%, whereas the average accuracies for the DTF with the PNN were 82.16% and 82.81% for 5- and 10-fold CV, respectively. The classification accuracies and other evaluation parameters for Case 2 are presented in Table 3.

From Table 2 and Table 3, it can be seen that, independent of the classifier, the classification based on PDC was better than that on the DTF. This was due to the fact that the PDC could eliminate the indirect effects of sources in the system [64]. Suppose that three sources—X1, X2, and X3—in a system are communicating such that X1 drives X2 while X2 drives X3. Thus, the connectivity between them should be X2 ← X1 and X3 ← X2, which will be correctly identified by the PDC. On the other hand, due to indirect effects [65], the DTF also suggests that X1 drives X3 (X3 ← X1), which is incorrect. As the number of sources increases in a system, these indirect connections also increase. Since there is no way to differentiate between direct and indirect connections in the DTF, the classification accuracy based on the DTF is less than that with the PDC.

The evaluation parameters for the different classification algorithms stated in Cases 1 and 2 were calculated in terms of the TPR (true positive rate), TNR (true negative rate), PPV (positive predictive value), FPR (false positive rate), and FNR (false negative rate). The probabilistic neural network achieved the maximum values of the TPR, TNR, and PPV when used with the PDC as well as the DTF for both 5- and 10-fold cross-validation. The maximum values for the FPR and FNR and the minimum values for the TPR, TNR, and PPV were recorded for the decision tree, which provided the lowest classification accuracy with both the PDC and DTF using 5- and 10-fold CV.

**Figure 7:**
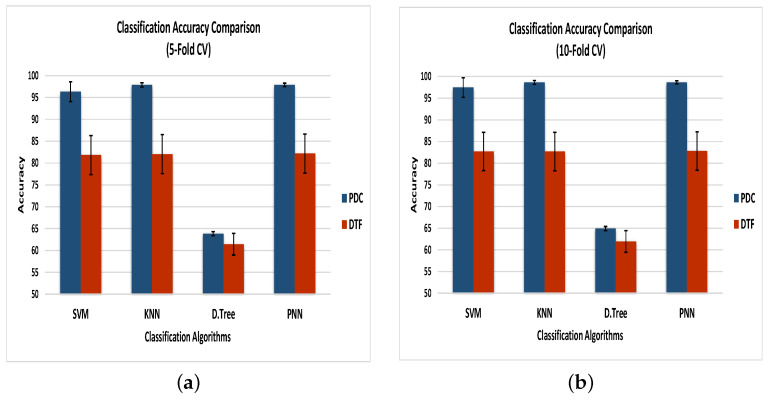
Classification accuracy of the four classifiers—SVM, KNN, decision trees, and PNN—based on the PDC and DTF as the feature sets when using (**a**) 5-fold CV and (**b**) 10-fold CV.

Figure 7 demonstrates the comparison of classification accuracies along with the errors of the classifiers using 5- and 10-fold CV, as described in Cases 1 and 2. Using the 5-fold cross-validation, the PNN outperformed all other classification algorithms by achieving average classification accuracies of 97.87% and 82.16% for the PDC and DTF, respectively, whereas the 10-fold CV resulted in enhanced maximum accuracies of 98.65% for the PDC and 82.81% for the DTF when using the PNN as a classification algorithm. On the other hand, the decision tree gave the lowest accuracy using the PDC and DTF for both 5- and 10-fold cross-validation. The error rate for each classifier using the DTF was greater than that of the classifiers when using the PDC. The minimum error was recorded for the PNN when using 10-fold CV, whereas the maximum was presented by the SVM when using 5-fold cross-validation.

As discussed earlier, the MI classification was performed for 91 healthy subjects, and the results presented above are the averages of all of the subjects. Among all 91 subjects, the PNN’s accuracy with the PDC varied between 94.01% and 98.28% for 5-fold CV and between 95.06% and 99.00% for 10-fold CV. In contrast, the PNN’s accuracy for the DTF varied between 70.66% and 92.95% for 5-fold CV and between 71.08% and 93.33% for 10-fold CV.

However, the standard deviation of the classification accuracy among the 91 subjects for each case provided information about the stability of the given classifiers. The standard deviation of the classification accuracy in each case is given in Table 4.

From Table 4, it can be seen that the PNN classifier had the lowest standard deviation for both Case 1 and Case 2, whereas the SVM classifier had the maximum standard deviation for both cases. Therefore, the PNN was proven to be the most stable classifier in both cases.

Although the PNN outperformed the other classification algorithms, there was a small difference between the prediction accuracies of the PNN and KNN. One of the major disadvantages of kNN is that the technique is not precise when calculating class probabilities with low values of k [66]. However, the PNN is an exclusive classifier, since a number of classifications can map every input pattern. The PNN’s main benefits include, an intrinsically parallel training process, no issues with local minima, a fast training procedure, and the assurance that the training structure converges on an optimum classifier as the size of the training set increases. Once the training samples have been added or deleted, no significant retraining is required. As a result, a PNN learns faster than neural networks and has already been shown to be successful in a range of tasks. A PNN is a supervised neural network that may be used for system categorization and pattern recognition based on these facts and benefits [67]. Another benefit of the PNN method is that probabilities for classification results may be immediately determined from a structural analysis. It is unlike other classification techniques, such as the SVM, which performs the process for calculating the probabilities associated with the classification results as a separate step after the model is created [68]. The computational cost of the proposed methodology is based on the k-fold cross-validation technique. It takes around 9.5 min on average to cross-validate each fold. However, the testing takes just a few seconds after the training procedure.

### 5.3. Comparison of the Proposed EC-Based MI EEG Classification Methods with Conventional Methods and Related Published Papers

We tested the prediction of the two-class MI EEG using the same 14-channel EEG dataset with several traditional feature extraction techniques, including the average power, root mean square, standard deviation, variance, entropy, discrete wavelet transform (DWT), and power spectral density (PSD). A comparison of the proposed and traditional methods is given in Table 5, which justifies the significance of the connectivity features compared to the traditional feature extraction techniques. Thus, it proves the hypothesis of achieving better results with the connectivity features.

In contrast with the proposed work in which brain connectivity analysis was utilized in the prediction of two-class motor imagery with the Physionet MI database, Sagee et al. [69] used wavelet decomposition for mu and beta rhythms with Naive Bayes and ANN classifiers to achieve the accuracies of 86.31% and 93.05%, respectively. Kim et al. [70] used the strong uncorrelating transform complex common spatial patterns (SUTCCSP) algorithm to extract features after obtaining the mu and beta bands through multivariate empirical mode decomposition (MEMD). The authors achieved an accuracy of 77.70% with a random forest classifier by using the extracted features. Dose et al. [71] extended the use of deep neural networks by incorporating subject-specific adaptation with transfer learning to get 86.49% accuracy for two-class MI prediction. Lun et al. [72] used a novel deep learning framework based on graph convolutional neural networks (GCNs) that learned the generalized features, and this achieved a classification accuracy of 88.57%. Qiu et al. [73] calculated the symbolic transfer entropy (STE) between electrode channels and constructed the brain networks of various cognitive behaviors of each participant by using the directed minimum spanning tree (DMST) algorithm. Finally, the spectral distribution set scoring (SDSS) method was used to recognize 69.35% of the labels. Carlos et al. [74] used a functional-connectivity-based graph method to acquire features and used PSD-based feature selection techniques to obtain 90% accuracy by using a linear discriminant analysis (LDA) classifier. Funda et al. [75] proposed a two-stage channel selection method and local transformation-based feature extraction for the classification of motor imagery/movement tasks and achieved a significant prediction of 95.95% by using KNN (see Table 6).

## 6. Conclusions

This study aimed to use the effective connectivity of the brain by considering inter-channel/region relationships during the imagination of left-/right-hand movements, which were determined by using the partially directed coherence (PDC) and directed transfer function (DTF). The PDC and DTF were then used as feature inputs for four types of machine learning (ML) algorithms for the classification of left and right motor imagery classes. A probabilistic neural network (PNN) based on the PDC features outperformed other PDC- and DTF-based ML algorithms. The PDC manifested its prediction superiority over DTF due to its ability to eliminate the indirect effects of sources in the system. The proposed framework solved a major disadvantage of conventional techniques by integrating a better knowledge of the brain’s neural patterns to improve the consistency and complexity, as well as by using multiple brain areas instead of just sensorimotor regions. The high classification accuracy of the left and right motor imagery based on the effective connectivity of the brain strongly supports the use of the PDC in BCI motor imagery applications. The use of graph theory for the identification of specific motor imagery patterns can be incorporated into the proposed technique to help people with motor disabilities by providing them with some reliable assistive technology, such as a brain-controlled wheelchair. However, there is a need for further testing on multiple classes (more than two), along with the optimization of the process in order to reduce the computational cost before the proposed technique can find its way in real-time BCI applications.

## Figures and Tables

**Table 1:** List of *p*-values of 105 significant connection pairs.

Pair	*p*-Value	Pair	*p*-Value	Pair	*p*-Value
T7←P7	0.000	CP3←Cz	0.025	C4←T8	0.022
T7←FC3	0.000	CP3←P4	0.000	CP4←P7	0.000
T7←P3	0.001	CP3←P8	0.000	CP4←FC3	0.000
T7←Fz	0.000	P3←FC3	0.000	CP4←C3	0.000
T7←Cz	0.011	P3←Fz	0.000	CP4←P3	0.003
T7←C4	0.000	P3←Cz	0.000	CP4←Cz	0.000
T7←CP4	0.008	P3←C4	0.024	CP4←FC4	0.005
T7←P4	0.000	P3←P4	0.019	CP4←C4	0.002
P7←P7	0.005	P3←P8	0.002	CP4←P4	0.000
P7←FC3	0.000	P3←T8	0.000	P4←P7	0.000
P7←Fz	0.000	Fz←P7	0.002	P4←FC3	0.000
P7←C4	0.017	Fz←P3	0.011	P4←C3	0.000
P7←P4	0.000	Fz←FC4	0.000	P4←CP3	0.000
P7←P8	0.000	Fz←CP4	0.010	P4←FC4	0.002
FC3←P7	0.000	Fz←P8	0.000	P4←C4	0.027
FC3←C3	0.003	Cz←CP3	0.005	P4←CP4	0.000
FC3←CP3	0.005	Cz←Fz	0.000	P4←P8	0.000
FC3←Cz	0.000	Cz←P4	0.000	P4←T8	0.003
FC3←CP4	0.001	Cz←P8	0.021	P8←P7	0.000
FC3←P4	0.047	Cz←T8	0.021	P8←FC3	0.000
FC3←T8	0.000	FC4←P7	0.000	P8←C3	0.000
C3←P7	0.000	FC4←C3	0.000	P8←C4	0.000
C3←FC3	0.020	FC4←CP3	0.000	P8←P4	0.000
C3←CP3	0.000	FC4←Fz	0.000	P8←P8	0.040
C3←Fz	0.023	FC4←CP4	0.000	P8←T8	0.001
C3←Cz	0.000	FC4←P4	0.000	T8←P7	0.000
C3←FC4	0.000	FC4←P8	0.002	T8←FC3	0.000
C3←C4	0.000	FC4←T8	0.000	T8←C3	0.029
C3←P4	0.000	C4←P7	0.000	T8←P3	0.000
C3←P8	0.022	C4←C3	0.000	T8←Fz	0.003
CP3←P7	0.000	C4←Cz	0.000	T8←Cz	0.000
CP3←FC3	0.000	C4←FC4	0.000	T8←C4	0.000
CP3←C3	0.000	C4←CP4	0.000	T8←CP4	0.000
CP3←CP3	0.024	C4←P4	0.013	T8←P4	0.000
CP3←Fz	0.036	C4←P8	0.000	T8←P8	0.000

**Table 2:** Performance in left/right MI EEG classification when using the PDC.

EC	k-Fold	Classifier	CA (%)	TPR (%)	TNR (%)	PPV (%)	FPR (%)	FNR (%)
PDC	5-Fold CV	SVM	96.30	95.49	97.19	97.37	2.81	4.51
		KNN	97.85	97.90	97.81	97.90	2.19	2.10
		D. Tree	63.85	64.57	63.10	64.89	36.90	35.43
		PNN	97.87	97.93	97.82	97.92	2.18	2.07
	10-Fold CV	SVM	97.45	96.98	97.96	98.08	2.04	3.02
		KNN	98.63	98.68	98.60	98.60	1.40	1.32
		D. Tree	64.92	65.58	64.21	66.00	37.79	34.42
		PNN	98.65	98.68	98.63	98.69	1.37	1.32

**Table 3:** Performance in left/right MI EEG classification when using the DTF.

EC	k-Fold	Classifier	CA (%)	TPR (%)	TNR (%)	PPV (%)	FPR (%)	FNR (%)
DTF	5-Fold CV	SVM	81.83	77.43	88.84	91.50	11.16	22.57
		KNN	82.04	82.38	81.68	82.51	18.32	17.62
		D. Tree	61.42	62.19	60.62	62.58	39.38	37.81
		PNN	82.16	82.64	81.65	82.49	18.35	17.36
	10-Fold CV	SVM	82.69	78.55	89.04	91.46	10.96	21.45
		KNN	82.67	83.02	82.34	83.14	17.66	16.98
		D. Tree	61.95	62.72	61.15	63.03	38.85	37.28
		PNN	82.81	83.27	82.33	83.13	17.67	16.73

**Table 4:** Standard deviation (SD) of the accuracy for the classification of MI EEG based on the PDC and DTF when using 5- and 10-fold cross-validation.

EC	Classifier	SD (%)		EC	Classifier	SD (%)	
		5-Fold	10-Fold			5-Fold	10-Fold
PDC	SVM	2.26	2.24	DTF	SVM	4.47	4.45
	KNN	0.48	0.44		KNN	4.46	4.46
	D.Tree	0.47	0.48		D.Tree	4.44	4.41
	PNN	0.39	0.34		PNN	2.47	2.50

**Table 5:** Comparison of the proposed method with the conventional feature extraction techniques in terms of classification accuracy (%).

Classifier	Proposed Features		Conventional Feature						
	PDC	DTF	F1	F2	F3	F4	F5	F6	F7
**SVM**	97.45	82.67	75.72	74.17	79.82	80.55	62.25	77.28	74.12
**KNN**	98.63	82.69	77.03	78.53	81.31	81.97	62.54	78.19	74.86
**D.Tree**	64.92	61.95	74.87	73.64	76.33	74.81	60.92	76.94	71.47
**PNN**	98.65	82.81	78.26	78.91	82.28	82.62	64.76	80.47	75.98

F1—Average power, F2—Root mean square, F3—Standard deviation F4—Variance, F5—Entropy, F6—DWT, and F7—PSD.

**Table 6:** Comparison of the classification accuracy with related papers that used the same dataset (the Physionet EEG motor imagery dataset).

Work	Year	Channels	Features	Classification Method	Accuracy (%)
Y. Kim et al. [70]	2016	14	Strong uncorrelating transform complex common spatial patterns (SUT-CCSP)	Random Forest	77.70
GS. Sagee et al. [69]	2017	64	Mu and beta rhythms	ANN	93.05
C. Filho et al. [74]	2017	64	FC-based graph method	LDA	90.00
H. Dose et al. [71]	2018	64	Raw EEG data	1D CNN	86.49
FK. Onay et al. [75]	2019	22	1D local transformation-based features	KNN	95.95
X. Lun et al. [72]	2020	64	Time-resolved EEG data	Graph CNN (GCNs)	88.57
L. Qiu et al. [73]	2020	64	symbolic transfer entropy (STE)	Directed minimum spanning tree (DMST)	69.35
**Proposed Work**	2021	14	Partial directed coherence (PDC)	**PNN**	**98.65**
			Directed transfer function (DTF)		**82.81**

## Data Availability

The databases used in this study are public and can be found at https://www.physionet.org/content/eegmmidb/1.0.0/ (accessed on 20 August 2021).
